# The Effect of Yoga on Health-Related Fitness among Patients with Type 2 Diabetes Mellitus: A Systematic Review and Meta-Analysis

**DOI:** 10.3390/ijerph19074199

**Published:** 2022-04-01

**Authors:** Rakhmat Ari Wibowo, Riskah Nurámalia, Herlin Ajeng Nurrahma, Eva Oktariani, Jajar Setiawan, Ajeng Viska Icanervilia, Denny Agustiningsih

**Affiliations:** 1 Department of Physiology, Faculty of Medicine, Public Health and Nursing, Universitas Gadjah Mada, Yogyakarta 55281, Indonesia; rakhmatari@mail.ugm.ac.id (R.A.W.); jajarsetiawan@ugm.ac.id (J.S.); denny_agustiningsih@ugm.ac.id (D.A.); 2 Department of Physiotherapy, Faculty of Nursing, Universitas Hasanuddin, Makassar 90245, Indonesia; 3 Department of Physiology, Faculty of Medicine, Sultan Agung Islamic University, Semarang 50112, Indonesia; herlinajengn@unissula.ac.id; 4 Faculty of Medicine, Universitas Abdurab, Pekanbaru 28291, Indonesia; eva.oktariani@univrab.ac.id; 5 Department of Radiology, Faculty of Medicine, Public Health and Nursing, Universitas Gadjah Mada, Yogyakarta 55281, Indonesia; ajeng.viska.i@ugm.ac.id or; 6 Department of Health Sciences, University Medical Center Groningen, University of Groningen, 9713 GZ Groningen, The Netherlands

**Keywords:** cardiorespiratory fitness, diabetes mellitus, exercise, yoga, muscle strength, physical fitness

## Abstract

Background: There is a need for a type of physical activity that could address the challenging cycle of physical inactivity, impaired health-related fitness, and type 2 diabetes mellitus (T2DM) conditions. Yoga could be one type of exercise to overcome the barriers to adhere to regular physical activity. The current study aimed to systematically review the effect of yoga on health-related fitness, including cardiorespiratory fitness, muscle strength, body composition, balance, and flexibility, among patients with T2DM. Methods: We systematically searched four databases and two registries (Pubmed, Scopus, Cochrane, Embase, WHO-ITCRP, and Clinicaltrials.gov) in September 2021, following a registered protocol on PROSPERO (CRD42022276225). Study inclusion criteria were T2DM patients with or without complication, yoga intervention as a single component or as a complement compared to other kinds of exercise or an inactive control, health-related fitness, and a randomized, controlled trial or quasi-experimental with control group design. The ROBINS-I tool and ROB 2.0 tool were used to assess the risk of bias in the included studies. A vote-counting analysis and meta-analysis computed using random effects’ models were conducted. Results: A total of 10 records from 3 quasi-experimental and 7 randomized, controlled trials with 815 participants in total were included. The meta-analysis favored yoga groups compared to inactive controls in improving muscle strength by 3.42 (95% confidence interval 2.42 to 4.43), repetitions of chair stand test, and improving cardiorespiratory fitness by 6.6% (95% confidence interval 0.4 to 12.8) improvement of baseline forced vital capacity. The quality of evidence for both outcomes was low. Conclusion: Low-quality evidence favored yoga in improving health-related fitness, particularly muscle strength and cardiorespiratory fitness, among patients with T2DM. Funding: All authors in this systematic review received no specific grant from any funding agency in the public, commercial, or not-for-profit sectors.

## 1. Introduction

Type 2 diabetes mellitus (T2DM) is one of the largest public health concerns leading to significant premature mortality and serious economic burden [1,2,3,4]. Evidence has shown that health-related fitness, such as cardiorespiratory fitness, muscle strength, and body composition, is an independent predictor of reduced quality of life, cardiovascular risks, and mortality among patients with T2DM [5,6,7,8,9]. Epidemiological studies found that patients with T2DM are frequently found to have low cardiorespiratory fitness and impaired muscle mass and strength as well as altered body composition [6,10,11,12]. This altered health-related fitness can be attributed to a pathological cycle of increased insulin resistance, vascular alteration, chronic inflammation, and lipid infiltration in patients with T2DM [13,14,15,16]. Therefore, intervention and therapy targeting this cycle in patients with T2DM is required to reduce morbidity and mortality, which then can improve their quality of life.

Strong evidence has shown the benefits of physical activity to health-related fitness [17]. However, most patients with T2DM did not adhere to physical activity recommendations [12]. Perceptions that exercise potentially exacerbates diabetes, feelings of inability to do exercise, and lack of facilities for carrying out exercise are among the most mentioned barriers to exercise among patients with T2DM [18]. Yoga is a mind–body exercise that is considered to be a suitable option for physical activity for patients with type 2 diabetes mellitus because of its low cardiovascular demands, low impact, simplicity, and easiness that could address the patients’ barriers to physical activity [19,20].

While yoga requires only light intensity during most of its session, it has still been found to provide health benefits for patients with T2DM since several of its poses during a session can result in moderate intensity [20,21]. A recent systematic review also found that yoga provides benefits for certain aspects of health-related fitness, including muscle strength, flexibility, and balance, as well as quality of life among elderly people [22]. However, the results from that review cannot be generalized to patients with T2DM since there are physiological differences among them. Thus, we conducted a systematic review to assess the effectiveness of yoga intervention compared to other exercise interventions and inactive controls on health-related fitness and quality of life for patients with T2DM.

## 2. Methods

A systematic review was conducted based on a registered protocol on PROSPERO (CRD42022276225), which was developed in advance of the review in accordance with guidelines from the Cochrane Collaboration and the Preferred Reporting Items for Systematic Reviews and Meta-Analyses 2020 Statement [23,24].

### 2.1. Inclusion Criteria

The inclusion criteria for studies were (1) population, studies with adult patients diagnosed with T2DM either with or without complications were included; (2) intervention and comparison, studies comparing yoga to either another exercise intervention or an inactive control or waiting-list control were included. Studies evaluating yoga as a combination with other exercises were included if there were comparators allowing evaluation of yoga as either a single component or a complement. Studies comparing one to another kind of yoga were excluded; (3) outcomes, only studies reporting at least one component of health-related fitness (cardiorespiratory fitness, muscle strength, body composition, balance, or flexibility) were included; and (4) type of study, studies with either a randomized, controlled trial (RCT) or a quasi-experimental with a control group design were included to anticipate insufficient number of RCTs addressing the health-related fitness outcomes. To be included in this review, health-related fitness outcomes must have been able to be assessed using objective measurements. We included studies that objectively measured cardiorespiratory fitness by either direct, indirect, maximal, submaximal, pulmonary functions or functional tests. Studies conducting muscle strength measurements by any instruments, such as the Oxford scale, dynamometer, or functional strength testing, were included. We included studies assessing body composition using magnetic resonance imaging, computed tomography, dual-energy X-ray absorptiometry, bioelectrical impedance analysis, and anthropometric measurements, including skin fold, waist circumference, hip circumference, or waist-to-hip ratio. Studies assessing flexibility or balance using any objective measurement were included.

### 2.2. Search Strategy

Four databases (Pubmed, Scopus, Cochrane, and Embase) and two registries (WHO-ICTRP and Clinicaltrials.gov) were searched by RAW and AVI from inception through September 2021. Search strategies were developed based on the population criteria using MeSH terms and free terms related to “Yoga” and “Type 2 Diabetes”. The complete search strategy used in each database is presented in the supplementary section (Supplement file S1). The outcome, comparator, and type of study were applied at the screening stage. The reference list of included studies and trial registries found during the database searches were also checked for additional relevant studies [25].

### 2.3. Study Selection

Having checked and removed duplicates, two reviewers (R.N., H.A.N.) conducted two stages of the screening process using the Rayyan software [26]. First, they screened independently titles and abstracts of all studies by categorizing them into “Yes”, ”No”, and “Maybe”. They only categorized studies that explicitly had different populations, types of study, interventions, and comparisons. They did not exclude abstracts that did not report health-related fitness as their outcomes since the majority of abstracts in biomedical research did not fully report all of their research outcomes [27,28]. Finally, they screened the full text of studies included in “Yes” and “Maybe” categories. The third reviewer (R.A.W.) facilitated discussion to resolve any disagreements in the first and second stages of the screening process. The reference list of included studies was checked by R.A.W.

Two reviewers (R.A.W., E.O.) developed and piloted a custom data extraction form (Supplement file S2). Descriptive and outcome data for all included studies were independently extracted by two reviewers (E.O., J.S.). Discussions facilitated by another reviewer (R.A.W.) were conducted to resolve discrepancies.

One reviewer (R.A.W.) assessed the risk of bias using the Cochrane Risk of Bias 2.0 tool for RCT and the risk of bias in non-randomized studies of interventions’ assessment (ROBINS-I) tool for quasi-experimental studies [29,30].

### 2.4. Synthesis Methods

We presented the narrative synthesis based on a vote-counting approach by categorizing the results of each outcome into three categories as follows: (1) statistically significant positive effects favoring yoga group, (2) statistically significant negative effects of the yoga intervention, and (3) no statistically significant difference between groups [31,32]. The results of the vote counting were based on the highest number of votes counted on each outcome. Then, meta-analyses were performed using the RevMan software for cardiorespiratory fitness, muscle strength, and body composition outcome since quantitative data from two or more studies were available and appropriate [32]. We combined groups from multiple intervention groups in one study to avoid double counts of the participants in the yoga group [32]. We multiplied mean values from one set of studies that had a scale with opposite direction to the common scale [32]. Since several studies used more than one instrument to measure an outcome, the most commonly reported outcome measures were included in the meta-analysis. For cardiorespiratory fitness, forced vital capacity (FVC) and a 6-min walk test were included in the meta-analysis, and chair stand tests were included for muscle strength outcome [33,34,35]. A random effects’ model was used since there was clinical heterogeneity resulting from variety in the yoga poses, frequency, session duration, and duration of the intervention [32]. Mean difference of the change of the chair stand test result from baseline between the intervention group and the control group (MD) was used because similar instruments were reported [32]. On the other hand, the standardized mean difference of the change of cardiorespiratory fitness from baseline was used because of the difference in instruments [32]. We obtained standard deviation (SD) of the change from baseline by imputing it from the *p*-value [32]. Meta-analysis of the final values of body composition using the standardized mean difference was conducted since the change scores of the body composition outcomes were not available in the primary studies and there were variabilities in the measure of body composition outcomes [32]. After that, we used the weighted mean difference and weighted standardized mean difference and then created forest plots to compute the effect size with 95% confidence intervals (CI) [32]. Weighted standardized mean differences were interpreted by expressing them into the most representative measurement instrument [30]. I^2^ statistics was used to assess statistical heterogeneity. Substantial heterogeneity was considered if there was I^2^ exceeding the threshold of 50% [30]. Subgroup analyses based on study design and duration of the intervention were conducted to explore heterogeneity among study results. Sensitivity analysis was conducted by excluding studies with a high risk of bias. The quality of evidence was evaluated using the Grading of Recommendations, Assessment, Development and Evaluation (GRADE) approach [32].

## 3. Results

**Figure 1:**
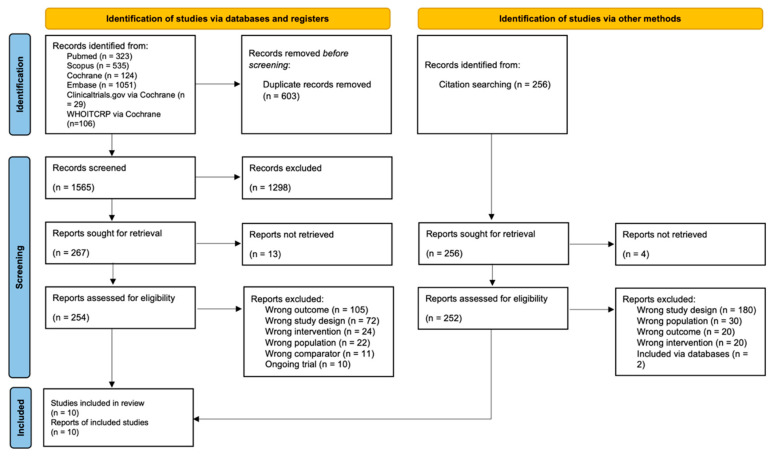
PRISMA flow diagram.

We identified 1117 records through database searches. Having conducted two stages of screening, we included 10 studies in the systematic review (Figure 1). Among the included studies, there were three quasi-experimental studies [36,37,38] and seven RCTs [39,40,41,42,43,44,45]. One quasi-experimental study assessed muscle strength, balance, and fall-related outcomes [37], one quasi-experimental study assessed body composition [36], and another quasi-experimental study assessed body composition and cardiorespiratory fitness [38]. On the other hand, one RCT assessed cardiorespiratory fitness, muscle strength, balance, and quality of life [41], another RCT assessed body composition and quality of life [44], another RCT only assessed cardiorespiratory fitness [39], and the remaining four only assessed body composition [40,42,43,45].

Cardiorespiratory fitness outcomes were measured using lung function tests assessing forced expiratory volume for 1 s (FEV1), FVC, FEV1/FVC ratio, slow vital capacity (SVC), peak expiratory flow rate (PEFR), and maximal voluntary ventilation (MVV); FVC, FEV1, and FEV1/FVC ratio were the most commonly reported measures of lung function. Since FVC ratio represented the degree of restrictive lung disease in patients with T2DM, which resulted in impaired cardiorespiratory fitness, FVC was chosen to be included in the meta-analysis using the standardized mean difference along with the distance in the 6-min walk test [16]. Among included studies, muscle strength was assessed by the chair stand test and the step-up test for lower extremity muscle strength and by the arm curl test for upper extremity muscle strength. Waist circumference, hip circumference, and waist-to-hip ratio were used to measure body composition among the included studies. Therefore, the standardized mean difference was used in the meta-analysis on body composition outcome. Balance was assessed using the Fullerton Advanced Balance (FAB) Scale, star excursion balance test, and single limb stance test. However, measures of balance were not included in the meta-analysis because of the lack of an included study.

Only two of the included studies were from the USA and UK [41,44]; the rest were from India. The number of participants in the included studies ranged from 18 to 160. The mean age of the participants ranged from 45 years to 60 years. Two studies only recruited female subjects, and another one did not report the proportion of each gender (Table 1).

Based on vote counting, the quasi-experimental studies favored yoga intervention compared to the inactive control in improving cardiorespiratory fitness, muscle strength, body composition, balance, and fall-related outcome (Table 2). While one RCT favored yoga intervention in improving muscle strength [42], the RCTs showed inconsistent effects of yoga intervention on cardiorespiratory fitness, body composition, and quality of life [39,40,41,42,43,44,45]. Quasi-experimental studies indicated that yoga provided at least similar benefits compared to other exercise interventions on muscle strength, body composition, balance, and fall-related outcome [36,37].

**Figure 2:**
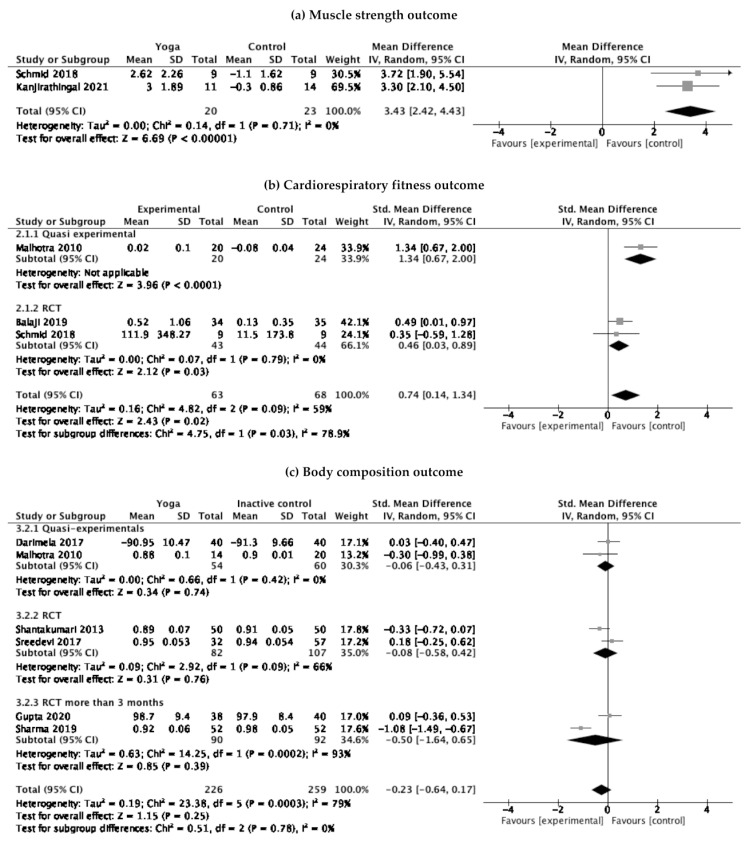
Forest plot for (**a**) muscle strength outcome, (**b**) cardiorespiratory fitness outcome, and (**c**) body composition outcome.

Two studies (one quasi-experimental and one RCT) assessing muscle strength, three studies (one quasi-experimental and two RCTs) assessing cardiorespiratory fitness, and six studies (two quasi-experimentals and four RCTs) assessing body composition were included in the meta-analyses. Regarding lower extremity muscle strength, yoga was found to be beneficial in improving 3.43 repetitions (95% CI 2.42 to 4.43) of the chair stand test (Figure 2). There was no significant heterogeneity on muscle strength outcome. Regarding cardiorespiratory fitness, yoga was found to be beneficial for improving FVC by 0.26 L (95% CI 0.05 to 0.47) (Figure 2). However, there was substantial heterogeneity. Having conducted subgroup analyses based on study design, we found that yoga still provided benefits without substantial heterogeneity by improving FVC by 0.16 L (95% CI 0.01 to 0.31), which was equivalent to a 6.6% (95% CI 0.4 to 12.8) improvement of the baseline FVC among the yoga group. No significant difference on body composition was found between the yoga and inactive control groups. While there was no heterogeneity among quasi-experimental studies, there were substantial heterogeneities among RCTs even after subgroup analyses based on the duration of the intervention. In sensitivity analysis excluding studies with a high risk of bias, there was still no significant effect of yoga on body composition outcomes (standardized mean difference = 0.14; 95% CI −0.17 to 0.45; *p* = 0.38). However, the heterogeneity was improved from I^2^ of 86% to 0%.

### 3.1. Risk of Bias

Most of the RCTs had “some concerns” of bias, while two RCTs had a “high risk” of bias and only one RCT had a “low risk” of bias (Table 3). Most of the bias in RCTs resulted from the randomization process, including unreported sequence generation and an allocation concealment process [31,37,40,42]. Most of the RCTS assessing the body composition outcome had bias resulting from the measurement of the outcome since they did not report the blinding process of the anthropometric measurement, which can be influenced by the assessor’s knowledge of the intervention received [42,43,44]. None of the quasi-experimental studies had a low risk of bias. Two quasi-experimental studies had a moderate risk of bias resulting from the imbalance of genders between the groups and the imbalance of the baseline characteristics, which could confound the effect of the intervention [36,37]. However, those confounders were not controlled using statistical analysis. Malhotra et al. [38] had a serious risk of bias due to several missing pieces of outcome data.

### 3.2. Quality of Evidence

We used GRADE tools to evaluate the quality of evidence for each outcome. Our meta-analysis on muscle strength had a narrow CI, which did not cross the minimal clinically important difference threshold in the chair stand test [46]. It also had direct evidence on yoga intervention among patients with T2DM, and consistent results reflected the absence of heterogeneity. However, there was a non-randomized study in the meta-analysis on muscle strength, with moderate risks of bias in the included studies. The publication bias could not be examined using a funnel plot because the number of available primary studies was less than 10 [32]. Therefore, the quality of evidence on muscle strength was low since there were two downgrades.

Having conducted a subgroup analysis based on the study design, the meta-analysis on cardiorespiratory fitness resulted from two RCTs that had a low risk of bias. The result was also consistent as reflected by the absence of heterogeneity. However, it did not have direct evidence on the effect of yoga on cardiorespiratory fitness among patients with T2DM since it came from the lung function results, which were not a primary test for cardiorespiratory fitness. In addition, the result was also imprecisely reflected from the wide CI crossing the minimum clinically important difference (MCID) of the FVC percent change [47]. Therefore, the quality of evidence on cardiorespiratory fitness was low. The quality of evidence on body composition was also low even after a subgroup analysis based on the study design. It resulted from four RCTs having some concerns and a high risk of bias.

## 4. Discussion

We provided low-quality evidence that yoga benefits muscle strength and cardiorespiratory fitness of patients with T2DM compared to the inactive control. In addition, a quasi-experimental study indicated that yoga could provide equal benefits on muscle strength compared to other exercise interventions. However, available evidence failed to show the benefits of yoga on body composition among patients with T2DM.

Yoga could be an alternative type of exercise for patients with T2DM because of the potential superiority of the yoga intervention addition to standard management alone and the equal benefits of yoga to other types of exercise on muscle strength and cardiorespiratory fitness. The improvement of 3.43 repetitions of the chair stand test was above the MCID of muscle strength. On the other hand, the benefit of yoga in improving cardiorespiratory fitness was imprecise since the confidence interval crossing the FVC change of 3% as the MCID. Our meta-analyses also failed to show a significant effect of yoga on body composition outcome. The results of the meta-analyses were in accordance with the findings across all of the included studies, showing a positive effect of yoga on muscle strength and an inconsistent effect on cardiorespiratory fitness and body composition. While yoga only required low metabolic intensity, the improvement in muscle strength could be caused by the isometric contraction during yoga poses, which could improve muscle strength and induce muscle hypertrophy regardless of intensity [20,48,49]. Forceful inspiration and expiration during yoga could be the cause of cardiorespiratory fitness improvement through the strengthening of respiratory muscle [50]. Therefore, yoga could be one potential type of exercise to address impaired muscle strength and possibly to improve cardiorespiratory fitness among patients with T2DM.

Our results were consistent with previous systematic reviews showing yoga benefits on muscle strength and cardiorespiratory fitness among the general population, elderly, and individuals with overweight or obesity [22,51,52,53]. Previous meta-analyses found a moderate effect size of yoga on muscle strength but there was heterogeneity [22,51]. The presence of medical conditions could be the source of clinical heterogeneity in the previous studies since our meta-analysis did not find any heterogeneity among patients with T2DM. Regarding the cardiorespiratory fitness outcome, the previous study also found a small effect size with a wide CI from three primary studies [51]. The uncertainty in cardiorespiratory outcome indicated the need to conduct more research on this outcome [54]. Our meta-analyses results were in accordance with previous systematic reviews showing that yoga and low-intensity exercise did not have a significant moderator effect on body fat percentage and waist circumference [43,44]. However, these results had wide CIs, resulting from the small number of primary studies. Therefore, more research should be conducted to examine the effect of yoga and other low-intensity exercise on body composition, particularly body fat percentage and waist circumference.

Having provided evidence systematically in accordance with the PRISMA guideline and fulfilling almost all of the A MeaSurement Tool to Assess Systematic Reviews version 2 (AMSTAR 2) checklist (Supplement file S3) [25], our reviews favored yoga intervention in improving health-related fitness among patients with T2DM. Our systematic review could not examine the publication bias due to the limited number of available studies. To minimize the publication bias, we also searched the Embase database, which covered gray literature such as conference proceedings [55]. To provide a higher quality of evidence, researchers should conduct more RCTs, undertaking and reporting the appropriate randomization and allocation concealment process. The results of our reviews should also be implemented cautiously in populations across genders since the majority of included studies recruited females.

## 5. Conclusions

A low quality of evidence favored yoga in improving health-related fitness compared to an inactive control, particularly muscle strength and cardiorespiratory fitness. For many patients with T2DM, a challenging cycle exists among a lack of physical activity, impaired health-related fitness, and T2DM conditions. Yoga as a light-intensity physical activity is a potential type of exercise to address T2DM patients’ barriers to physical activity. More high-quality RCTs are needed to provide a higher quality of evidence.

## Figures and Tables

**Table 1:** Characteristics of included studies.

Study id Country Funding Source	Study Design	Participants (Number, Mean Age (SD), Gender Proportion, Presence of Diabetic Complication)	Intervention Characteristics (Type, Frequency, Session Duration, Length of Intervention)	Control Group(s)	Outcome Measures
Darimela (2017), India, Funding not stated [36]	Quasi- experimental	*n* = 160, age range 36–48, 100% female, complication not described	Hatha yoga, up to 60–70 min per session, frequency not described, 6 months	1. Active control: exercise 2. Active control: walking exercise 3. Inactive control	Body composition: hip circumference
Kanjirathingal (2021), India, MGM School of Physiotherapy, MGM Institute of Health Sciences, Navi Mumbai, India [37]	Quasi- experimental	*n* = 35, mean age (SD): yoga group = 55.5 (7), mean age (SD): balance exercise group = 58.7 (5.6), mean age (SD): control group = 57.7 (6), 51.4% female, diabetic peripheral neuropathic pain	Hatha yoga, 3 times a week, 1 h per session, 12 weeks	1. Active control: usual care + balance exercise 2. Inactive control: usual care + wait-list control	Muscle strength: chair stand test, step-up test; balance: star excursion balance test, single limb stance test; fall-related outcome: modified fall efficacy scale
Malhotra (2010), India, Funding NA [38]	Quasi- experimental	*n* = 62, age range = 30–60, gender not available, complication not described	Hatha yoga, every day, 30–40 min per session, 40 days	1. Inactive control: usual care + mild exercise advice	Cardiorespiratory fitness: lung function test (slow vital capacity, forced expiratory volume for 1 second, peak expiratory flow rate, maximal voluntary ventilation, forced vital capacity) body composition: waist-to-hip ratio
Balaji (2019) India, Sri Balaji Vidyapeeth funds the CYTER and all of its activities in yoga therapy, education, and research [39]	RCT	*n* = 72, mean age (SD) = 49.6 (5.88), 31.9% female, diabetic lung function	Hatha yoga, thrice a week, 60 min per session, 4 months	1. Inactive control: Usual care + advice on diet	Cardiorespiratory fitness: Lung function test (Forced expiratory volume for 1 second, forced vital capacity, forced expiratory volume for 1 second/forced vital capacity ratio)
Gupta (2020), India Centre for Integrative Medicine and Research, All India Institute of Medical Science [40]	RCT	*n* = 81, mean age (SD) = 50.6 (8.5), 44.4% female, 42% were on blood pressure medication, 44.4% were on lipid-lowering medication	Integrated yoga; 45 min/session; 3 classes/week for the first 2 weeks, 2 classes/week for the next 2 weeks, and 1 class/month for the last 3 months; 4 months	Inactive control: dietary and walking advice	Body composition: waist circumference
Schmid (2018) USA, Colorado State University Prevention Research Center [41]	RCT	*n* = 18, mean age (SD) = 54.95 (9.94), 66.67% female, diabetic peripheral neuropathic pain	Hatha yoga, twice a week, duration not available, 8 weeks	1. Inactive control: usual care + wellness education	Cardiorespiratory fitness: 6-min walk test; muscle strength: upper extremity strength (chair stand test), lower extremity strength (arm curl test); balance: The Fullerton Advanced Balance Scale Quality of life: Rand 36-Item Health Survey
Shantakumari (2013), India, Funding NA [42]	RCT	*n* = 100, mean age = 45, 48% female, dyslipidemia	1 h/session, daily, 3 months	Inactive control	Body composition: waist-to-hip ratio
Sharma (2020), India, Rajasthan University of Health Sciences [43]	RCT	*n* = 104, age range = 30–65, 45.19% female, dyslipidemia	40 min/session, 5 days/week, 6 months	Inactive control	Body composition: waist-to-hip ratio
Skoro-Kondza (2009), UK, Novo Nordisk Research Foundation [44]	RCT	*n* = 59, mean age (SD) = 60 (10), 61.02% female, type 2 diabetes mellitus without complication	90 min/session, 2 days/week, 12 weeks	Inactive control: lifestyle leaflet and advice + waiting list yoga	Body composition: waist-to-hip ratio; quality of life: audit of diabetes-dependent QoL
Sreedevi (2017) India, Fogarty International Centre, National Institutes of Health [45]	RCT	*n* = 124, mean age (SD)= 51.9 (7.3); 100% female, dyslipidemia	60 min/session, 2 days/week, 3 months	1. Inactive control: standard advice on diet and exercise 2. Inactive control: peer-support on management of diabetes, diet, and exercise	Body composition: waist-to-hip ratio

**Table 2:** Vote-counting results.

Study id, Design	Cardiorespiratory Fitness	Muscle Strength		Body Composition		Balance		Fall-Related Outcome		Quality of Life
	vs. Inactive Control	vs. Active Control	vs. Inactive Control	vs. Active Control	vs. Inactive Control	vs. Active Control	vs. Inactive Control	vs. Active Control	vs. Inactive Control	
Darimela (2017), Quasi-experimental [36]				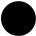	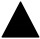					
Kanjirathingal (2021), Quasi-experimental [37]		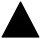	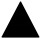			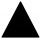	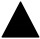	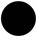	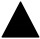	
Malhotra (2010), Quasi-experimental [38]	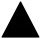				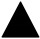					
Balaji (2019), RCT [39]	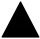									
Gupta (2020), RCT [40]					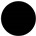					
Schmid (2018), RCT [41]	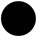		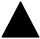							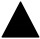
Shantakumari (2013), RCT [42]					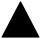					
Sharma (2020), RCT [43]					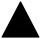					
Skoro-Kondza (2009), RCT [44]					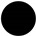					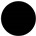
Sreedevi (2017), RCT [45]					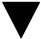					

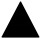
 statistically significant favoring yoga; 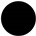 no statistically significant difference; 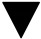 statistically significant favoring comparator.

**Table 3:** Risk of bias assessment.

ROB 2.0	Randomization Process	Deviation from Intended Intervention	Missing Outcome Data	Measurement of the Outcome	Selection of the Reported Results	Overall Bias		
Balaji 2019 [39]	Some concerns	Low	Low	Low	Low	Some concerns		
Gupta 2020 [40]	Low	Low	Low	Low	Low	Low		
Schmid 2018 [41]	Some concerns	Low	Low	Low	Low	Some concerns		
Shantakumari (2013) [42]	Some concerns	Low	Low	High	Low	High		
Sharma (2020) [43]	High	Low	Low	High	Low	High		
Skoro-Kondza (2009) [44]	Low	Low	Low	Some concerns	Some concerns	Some concerns		
Sreedevi (2017) [45]	Some concerns	Low	Some concerns	Low	Low	Some concerns		
**ROBINS-I**	**Confounding**	**Selection of participants**	**Classification of intervention**	**Deviation from intended intervention**	**Missing data**	**Measurement of outcome**	**Selection of reported results**	**Overall bias**
Darimela (2017) [36]	Moderate	Low	Low	Low	Low	Moderate	Low	Moderate risk of bias
Kanjirathingal (2021) [37]	Moderate	Low	Low	Low	Low	Low	Low	Moderate risk of bias
Malhotra (2010) [38]	Low	Low	Low	Low	Serious	Low	Low	Serious risk of bias

## Data Availability

No new data were created or analyzed in this study. Data sharing is not applicable to this article.

## Supplementary Materials

The following supporting information can be downloaded at https://www.mdpi.com/article/10.3390/ijerph19074199/s1. Supplementary file S1: Search Strategy; Supplementary file S2: Blank Data Extraction Form SysRev Yoga; Supplementary file S3: PRISMA Checklist.
