# Supplementary material for: The Effect of Yoga on Health-Related Fitness among Patients with Type 2 Diabetes Mellitus: A Systematic Review and Meta-Analysis
Source: Int J Environ Res Public Health. 2022 Apr 1;19(7):4199. doi: 10.3390/ijerph19074199 (PMC8998732; doi:10.3390/ijerph19074199)
Supplement: Supplementary file 1 [file ijerph-19-04199-s001.zip › Supplementary file 1 Search Strategy.pdf]

Search Strategy for Pubmed:
(YOGA OR YOGA[MeSH Terms] OR mind body exercise[Mesh terms] OR YOGIC) AND (NIDDM OR Diabetes Mellitus, Type 2[MeSH Terms] OR Diabetic)

Search Strategy for Scopus:
(TITLE-ABS-KEY(YOGA OR YOGIC OR YOGI OR (MIND-BODY AND EXERCISE) OR (MIND AND BODY AND EXERCISE)) AND TITLE-ABS-KEY(DIABETES OR NIDDM OR DIABETIC))

Search Strategy for Cochrane:
(YOGA OR YOGIC OR YOGI OR (MIND-BODY AND EXERCISE) OR (MIND AND BODY AND EXERCISE)) AND (DIABETES OR NIDDM OR DIABET OR DIABETIC)

Search Strategy for Embase
1= yoga/
2= mind-body therapies
3= (yoga or yogi or yogic).mp
4= Diabetes Mellitus, Type 2/
5= (NIDDM or diabetes or diabet or diabetic).mp
6= 1 or 2 or 3
7= 4 or 5
8= 6 and 7
